# Hydrogel models of pancreatic adenocarcinoma to study cell mechanosensing

**DOI:** 10.1007/s12551-024-01265-8

**Published:** 2024-12-18

**Authors:** M Walker, JP Morton

**Affiliations:** 1 Centre for the Cellular Microenvironment, Advanced Research Centre, 11 Chapel Lane, James Watt School of Engineering, University of Glasgow, Glasgow, G11 6EW UK; 2 Cancer Research UK Scotland Institute, Garscube Estate, Switchback Rd, Glasgow, G61 1BD UK; 3 School of Cancer Sciences, University of Glasgow, Garscube Estate, Switchback Rd, Glasgow, G61 1QH UK

**Keywords:** Pancreatic adenocarcinoma, Mechanobiology, Hydrogels, Biomedical engineering, Cancer biology, Biomaterials

## Abstract

Pancreatic adenocarcinoma (PDAC) is the predominant form of pancreatic cancer and one of the leading causes of cancer-related death worldwide, with an extremely poor prognosis after diagnosis. High mortality from PDAC arises partly due to late diagnosis resulting from a lack of early-stage biomarkers and due to chemotherapeutic drug resistance, which arises from a highly fibrotic stromal response known as desmoplasia. Desmoplasia alters tissue mechanics, which triggers changes in cell mechanosensing and leads to dysregulated transcriptional activity and disease phenotypes. Hydrogels are effective in vitro models to mimic mechanical changes in tissue mechanics during PDAC progression and to study the influence of these changes on mechanosensitive cell responses. Despite the complex biophysical changes that occur within the PDAC microenvironment, carefully designed hydrogels can very closely recapitulate these properties during PDAC progression. Hydrogels are relatively inexpensive, highly reproducible and can be designed in a humanised manner to increase their relevance for human PDAC studies. In vivo models have some limitations, including species-species differences, high variability, expense and legal/ethical considerations, which make hydrogel models a promising alternative. Here, we comprehensively review recent advancements in hydrogel bioengineering for developing our fundamental understanding of mechanobiology in PDAC, which is critical for informing advanced therapeutics.

## Pancreatic cancer biology

### PDAC: current challenges

Pancreatic adenocarcinoma (PDAC) is one of the leading causes of cancer-related death worldwide; in the USA alone, PDAC now has the third highest mortality rate, overtaking breast cancer, and is projected to overtake colorectal cancer before 2040, which would make it second only to lung cancer (Halbrook et al. 2023; Rahib et al. 2014; Rahib et al. 2021). One of the primary factors that leads to poor prognosis for PDAC patients is a failure to detect the disease until it is very advanced and often metastatic. Symptoms are often vague before this stage with no single attributable risk factor for most patients (Kleeff et al. 2016; Petersen 2016). The difficult-to-access anatomical location of the pancreas makes routine screening approaches challenging, and those eligible for surgery (only 15–20% after diagnosis) must be in robust health to withstand the operation, which is associated with significant morbidity and mortality (Halbrook et al. 2023; Kleeff et al. 2016). Even in those patients where surgical resection is possible, recurrence is common, and current chemotherapies offer limited survival benefits. There is a clear clinical need for both improved therapeutic options and novel, non-invasive approaches to diagnose PDAC at earlier stages for improving patient outcomes.

### The transition from healthy pancreas to PDAC

The healthy pancreas is composed of both exocrine and endocrine tissues that operate independently to regulate digestion and control glucose homeostasis through insulin and glucagon secretion. The endocrine cellular compartments are organised into discrete units called islets of Langerhans, and the exocrine pancreas contains clusters of acinar cells forming functional units termed acini. Acinar cells comprising these acini contain numerous vesicles containing various inactive enzymes, namely zymogen. Zymogen is secreted through a network of ducts, which ultimately coalesce to form the main pancreatic duct, which drains into the duodenum. Zymogen activation typically occurs only after reaching the duodenal lumen (Chu et al. 2007).

Pancreatitis is recognised as a precursor pre-tumour inflammatory phase, which marks critical stages during the development of PDAC. Pancreatitis occurs in two main forms, acute and chronic; the former involves a sudden presentation of symptoms, which are short-lived, and the latter relates to pancreatic damage over a longer time period. Acute pancreatitis is a common inflammatory disorder of the pancreas that causes severe abdominal pain and organ dysfunction which has 30–40 cases per 100,000 population per year and is a significant economic burden per person (Petrov and Yadav 2019; Andersson et al. 2013). This involves inappropriate activation of zymogen from acinar cells, resulting in tissue autodigestion, cytokine release and inflammation, which trigger acute pancreatitis. Acute pancreatitis can develop into a chronic form with common risk factors associated, including alcohol use, smoking and genetic predisposition, which has an extremely high mortality rate of up to 50% within 20–25 years (Steer Michael et al. 1995). A characteristic pathological marker of chronic pancreatitis is hypersecretion of specific proteins, such as Lithostathin, from acinar cells in the absence of increased fluid or bicarbonate secretion from duct cells, which gives rise to plugs and intraductal stones through precipitate formation (Steer Michael et al. 1995). Prevention or reduction of acute pancreatitis is essential to halt the progression towards chronic pancreatitis, which is a significant risk factor for PDAC (Szatmary et al. 2022). Some diagnostic markers for pancreatitis, such as human pancreatic lipase, have already been identified and play a critical role in clinical testing and disease treatment (Moridani and Bromberg 2003; Shi et al. 2017). Despite this, limitations of established biomarkers are that they require several preparative steps specialised equipment and, critically, are not conducive to early diagnosis (Zhou and Li 2015; Kumar et al. 2023; Miao et al. 2022). From a therapeutic perspective, acute pancreatitis requires nutritional support, prophylactic antibiotics and rehydration, whereas chronic pancreatitis is managed by lifestyle alterations regarding alcohol and smoking abstinence, pain control and replacement of pancreatic insufficiency or mechanical drainage of obstructed pancreatic ducts; unfortunately, all approaches lack long-term effectiveness (Zhou et al. 2024). From a diagnostic and therapeutic standpoint in pancreatitis, there is a clear need to develop novel approaches due to the limitations of current practices.

As well as pancreatitis, pancreatic intraepithelial neoplasias (PanINs) are also recognised as one of the best characterised and most frequent precursor lesions of PDAC; they are non-invasive microscopic lesions that are undetectable by radiological examination and exhibit differing degrees of mucin levels and abnormal cell morphologies (Matsuda et al. 2017). PanINs are preceded by acinar-to-ductal metaplasia (ADM), which relates to pancreatic acinar cells dedifferentiating into ductal-like cells. Acinar cells lose their characteristic shape and function and adopt a ductal-like cell morphology, which involves changes in the expression of genes that control cell differentiation, proliferation and survival; this cellular plasticity within the pancreas is exploited in tumorigenesis and can lead to the progression of PanINs and PDAC (Marstrand-Daucé et al. 2023). Alterations in the mechanical microenvironment during pancreatitis and PanINs could be pivotal in regulating the early stages of PDAC through ADM-mediated events. Indeed, previous studies have performed measurements on these stages of disease progression and shown distinct differences in their mechanical properties, which could be critical in regulating abnormal cell responses (Rice et al. 2017; Rubiano et al. 2018). Modelling these phases of PDAC progression could be highly informative for advancing diagnostic and therapeutic targets and thus expanding the therapeutic window for early clinical intervention.

Despite the importance of pancreatitis and PanINs as early prognostic markers for PDAC, very little is known about these microenvironments, particularly from a mechanobiology perspective, with significantly more focus being drawn towards PDAC tissue. As well as PDAC tissue, it is key to understand the role of biophysics in pancreatitis and PanINs for identifying novel diagnostic and therapeutic targets to expand the therapeutic window and improve clinical outcomes.

### Extracellular matrix (ECM)

The extracellular matrix (ECM) within the pancreatic microenvironment consists of many biomechanical and biochemical cues that regulate cell behaviour (Wang et al. 2022). This microenvironment is incredibly dynamic and requires a delicate balance of many factors to maintain tissue homeostasis (Hellewell and Adams 2016). Biomechanical cues include factors such as elasticity, viscosity and external tensional/compressive forces; collectively, these regulate key mechanosensitive signalling pathways of cells (Mecham 2012). Key biochemical components include proteins such as collagen, fibronectin, elastin and hyaluronic acid (HA), which contain bioactive motifs, such as integrin-binding sites, to facilitate processes like adhesion and migration (Humphrey et al. 2014).

The stromal transition from healthy pancreatic tissue to PDAC is a highly dynamic process involving a variety of cell types. In the normal exocrine pancreas, the pancreatic ductal epithelium is distinct from connective tissue, which includes fibroblasts and ECM, by the physical presence of a basement membrane. Adjacent to ducts are the acini, which are surrounded by pancreatic stellate cells (PSCs), which are a specialised mesenchymal cell. At intermediate PanIN stages, the ductal epithelium is reshaped to contain columnar mucin-containing cells that exhibit nuclear atypia. The basement membrane around these lesions remains intact, while alterations within the surrounding stromal tissue are seen, including fibroblastic and early vascular proliferation. Changes are also evident in the exocrine pancreas, which resembles pancreatitis-like alterations, including ADM and PSC activation. In PDAC, the basement membrane is breached, which allows tumour cell invasion into the surrounding pancreatic parenchyma. Significant dysregulation of ECM cues occurs in PDAC due to a highly fibrotic stromal response known as desmoplasia. Desmoplasia arises from reprogrammed cells in the tumour microenvironment (TME), such as cancer-associated fibroblasts (CAFs), that secrete abnormal quantities of ECM proteins, which forms a dense connective tissue network that is dramatically altered mechanically (Kai et al. 2019; Yeldag et al. 2018). It is thought that this dynamic alteration of ECM composition drastically alters the mechanosensing of cells as the microenvironment alters during the progression towards PDAC. Altered cellular mechanosensing causes them to abnormally differentiate towards disease phenotypes and behave in a manner that promotes chemoresistance, tumour progression and metastasis (Mierke 2024).

### Cell mechanosensing during PDAC

Cells respond to the changes in ECM mechanics resulting from desmoplasia in PDAC by altered interactions of force-sensing integrins. These begin the cascade of converting mechanical signals into biochemical signals that trigger alterations in cell behaviour, for instance, through focal adhesion kinase signalling (Duscher et al. 2014; Jiang et al. 2016). Indeed, integrin-β1 has been specifically implicated in PDAC; in the *Ptf1a-Cre; Kras*^LSL−G12D/+^ mouse model, the introduction of an integrin-β1 mutation that recapitulates tension-dependent integrin clustering and focal adhesion signalling, resulted in accelerated PDAC progression that coincided with elevated inflammation, fibrosis, and altered immune infiltrates (Laklai et al. 2016). Lysyl oxidase serves to crosslink collagen within the ECM, thereby altering mechanics and has been shown to promote invasive cancer cell behaviour through integrin-associated mechanosignalling (Levental et al. 2009).

Some key molecular players involved in mediating cell mechanosensing in PDAC are YAP/TAZ (yes-associated protein 1/transcriptional coactivator with PDZ-binding motif), Piezo1, integrin-linked kinase (ILK) and Rho-associated kinase (ROCK). YAP is perhaps the most widely investigated mechanosensitive protein and has been identified as a potent oncogene that is amplified in various cancers (Piccolo et al. 2023). Piezo1 is a mechanosensitive ion channel that is stretch-activated upon changes in mechanical perception; this regulates Ca^2+^ signalling, which is essential for key cellular processes and has been implicated in cancer when dysfunctional (Zhao et al. 2022). ILK is a multifunctional molecular actor in cell–matrix interactions, cell adhesion and anchorage-dependent cell growth, which has been associated with cancer (Górska and Mazur 2022). ROCK serine/threonine kinases are key regulators of the actomyosin cytoskeleton; their main purpose is to promote contractile force generation, which regulates intracellular tension and has been implicated in various cancers (Rath and Olson 2012).

### Modelling PDAC biomechanics: considerations and strategies

Developing in vitro models that effectively recapitulate changes in biomechanics observed during PDAC progression is fundamental to enhancing our understanding of the disease and identifying novel biomarkers and therapeutic targets. Hydrogels, as water-swollen polymer networks, are highly effective biomaterials for mimicking the biophysical and biochemical properties of the native ECM. Various natural and synthetic materials are often utilised to fabricate hydrogels, which have pros and cons.

Natural matrices, such as collagen, often retain naturally occurring bioactive sites within the ECM, such as integrin-binding and protease-cleavable sites, to allow cell attachment and remodelling, respectively, and support high biocompatibility; however, they are often more challenging to mechanically manipulate (Lee and Mooney 2001; Walker et al. 2021; Asadishekari et al. 2022). Collagen is an advantageous natural hydrogel material that has been used to fabricate hydrogels discussed in this review in the context of PDAC. Collagen has clear advantages for modelling tissue implicated in PDAC because it is the most abundant protein within the body, particularly collagen I, and is a key component of the natural ECM; despite its advantages, collagen is a relatively expensive material with batch-to-batch variability and can lead to undesired immunogenic reactions (Gómez-Guillén et al. 2011). Additionally, collagen hydrogels suffer from low mechanical tuneability, which may be challenging to model the increased mechanical values seen in PDAC tissue. Additionally, the lack of control over the presentation of bioactive sites means it can be challenging to decouple the role of biochemical from mechanical cues on cell response. Gelatin is a denatured version of collagen and has been widely used in PDAC studies discussed in this review, which offers some advantages over collagen-based materials. While gelatin retains the bioactive properties of collagen, it has reduced cost and lower adverse immune side effects (Gómez-Guillén et al. 2011). Another natural matrix that is used in this review for PDAC studies is Matrigel; this is perhaps the gold standard material used for 3D culture studies due to its high biocompatibility and applicability to a range of cell types. Matrigel is an assortment of ECM proteins derived from Englebreth-Holm-Swarm tumours in mice; the availability of many naturally occurring bioactive sites is an ideal matrix for recapitulating the biochemistry of the native cellular microenvironment during PDAC progression (Kleinman and Martin 2005). However, Matrigel is very poorly defined and has been associated with high variability in experimental results (Hughes et al. 2010). HA has significant implications in pancreatic cancer through its interactions with CD44 and has been associated with pancreatic cancer cell growth (Mattheolabakis et al. 2015; Kim et al. 2021). Alternative splice variants of CD44 have been identified as PDAC markers, which could be pivotal in altered functional cell processing during HA-CD44 interactions (Sato et al. 2016). In the context of modelling PDAC progression, HA has been incorporated into hydrogels in studies discussed within this review, which is promising given its role in regulating cell response within the ECM and can be mechanically tuned within matrices by incorporating specific crosslinking chemistries. However, a limitation of using HA is a lack of integrin-binding sites, which means functionalisation is required to allow cell adhesion. Crosslinkers used to alter the physical properties of the hydrogels may result in cell toxicity, which could narrow down the types and concentrations of crosslinkers that are incorporated (Burdick and Prestwich 2011).

Synthetic matrices can cause issues with biocompatibility but are generally much more mechanically tuneable and are biochemically inert to provide a blank canvas that can be modified with bioactive ligands for cells to interact with; in this way, synthetic options allow users to decouple mechanical from biochemical cues for a more detailed understanding of cell response (Lee and Mooney 2001; Walker et al. 2021; Walker et al. 2024). Perhaps the most widely used synthetic hydrogel material used in PDAC mechanobiology studies is polyacrylamide due to its simple preparation, relatively inexpensive, high reproducibility and mechanical tuneability; this has led to seminal methodological studies being published on the fabrication of polyacrylamide gels with highly controllable mechanics (Tse and Engler 2010). Additionally, since polyacrylamide is biochemically inert, this means that it cannot support cell attachment or growth alone and requires chemical modification to introduce bioactive moieties; therefore, polyacrylamide acts as a blank canvas for functionalisation with controlled densities of ligands to recreate desired ECM compositions during different stages of PDAC progression. Perhaps the main drawback of polyacrylamide is its lack of 3D culture suitability over longer periods due to the toxicity of precursor components; polyacrylamide also lacks biodegradability, which limits the ability of cells to remodel the environment (Caliari and Burdick 2016). Despite this, many key studies have been performed in 2D using polyacrylamide, which is discussed in this review, to provide great insight into mechanobiological responses during PDAC progression. Other synthetic materials, such as polyethylene glycol and peptide-based gels, have been used in PDAC studies discussed in this review, which can permit 3D culture and overcome the issues associated with 2D materials like polyacrylamide. These materials are generally biochemically inert and require modification with bioactive groups to support cell growth and offer similar advantages to polyacrylamide; however, consideration must still be taken when using certain crosslinking chemistries, which may be harmful to cells; also, peptide-based gels can suffer from low mechanical stability (Chen and Zou 2019; Liu et al. 2019; Zhu 2010).

As well as different hydrogel materials, a variety of cell types have been utilised for hydrogel mechanosensing research in PDAC, and it is critical that differences between these are outlined. Many studies utilise cell lines for their studies, which have advantages due to their more controllable nature and consistency of phenotype. Many of these cell lines are used to study epithelial-to-mesenchymal transition (EMT), which is a characteristic marker in PDAC; however, these cells often exist in different states of differentiation and thus have differing degrees of EMT potential. For instance, PANC-1 and Suit2 cells are discussed in this review; PANC-1 cells display a more mesenchymal phenotype, whereas Suit2 cells display a more epithelial phenotype (Shichi et al. 2022). Therefore, for studies into EMT, Suit2 cells would be more appropriate, given their tendency to differentiate from epithelial to mesenchymal cells. Additionally, despite morphological consistencies in 2D culture, when cells are cultured in 3D as organoids within hydrogels, epithelial PDAC cells form small spheres with coated cells on the surface, while mesenchymal PDAC cells form spheres that are loosely bound together; these features significantly influence their proliferation and anticancer drug responses (Shichi et al. 2022). Cells derived from genetically modified mouse models have also been employed, which have the advantage of being exposed to all the cues of the PDAC microenvironment within a complex, multicellular system. However, there are drawbacks to this approach, including the species-to-species variability between humans and mice, and thus, the relevance of the findings from these models, high mouse-to-mouse variability and the associated financial and legal requirements of working with mice models. These are all considerations that researchers should consider when choosing which cell type to use for their studies and the dimensionality of their system given its influence on cell response.

When considering early prognostic markers of PDAC, pancreatitis and PanIN are key predictive events that have been explored in hydrogel research. In terms of pancreatitis, some studies have been performed using hydrogels for diagnostic and treatment purposes; for instance, Ping et al. developed a diagnostic platform using a liquid crystal sensing device, which incorporates a gelatin hydrogel containing cetyltrimethylammonium bromide that is released and detected upon hydrogel degradation by pancreatic proteases giving rapid biomarker detection faster than conventional methods (Ping et al. 2021). In terms of pancreatitis treatment, decellularized ECM hydrogels derived from mesenchymal stem cells exhibit significant improvements in pancreatic tissue inflammation and fibrosis, as well as high cellular compatibility and biocompatibility in an induced rat pancreatitis model (Kojima et al. 2023). In terms of PanIN, Rice et al. developed 2D polyacrylamide models and revealed that the increased stiffness associated with PanIN resulted in a more pathological cellular phenotype of epithelial cells (Rice et al. 2017). Despite some interesting applications of hydrogels in pancreatitis and PanIN research, very little has been conducted exploring the role of the mechanical microenvironment on cellular mechanosensing. Given the critical role of mechanobiology in PDAC progression, it would be highly informative to explore its role in these early-stage pathologies for novel insights into expanding the therapeutic window of PDAC.

In this review, we summarise the latest works focusing on designing hydrogels for biomechanical studies into cell mechanosensing in PDAC. Specifically, we focus on how mechanics regulate key events in PDAC progression that are described in recent literature; these events are differentiation, chemoresistance and metastasis. We have ordered these sections as listed here to disseminate the information in a logical order, which describes events during PDAC progression; abnormal differentiation events, which give rise to primary tumours and generally precede chemoresistance and metastasis, are first discussed. Chemoresistance is then introduced as a phase, which begins once primary PDAC tumours with a dense stromal barrier are established. Finally, we discuss metastasis to describe migration towards secondary sites. Within these sections, we highlight aspects such as the signalling events and molecular players involved, the types of mechanical properties that cells respond to and the cellular behaviours that are studied.

## Hydrogel models to study mechanosensitive cell behaviour in PDAC

### Differentiation

One of the main cellular events that drives PDAC progression is the differentiation of cells from healthy to disease phenotypes. This process largely occurs in pancreatic epithelial cells, which are reprogrammed to exhibit more aggressive, migratory and invasive tendencies, but also in immune cells, and in fibroblasts, which secrete and remodel abnormal amounts of ECM (Malinova et al. 2021). During early abnormal differentiation events, the primary TME begins to establish, which is a key initial phase in PDAC progression.

EMT is one of the hallmark differentiation events in PDAC, which involves epithelial cells losing their cell–cell polarity and becoming more migratory and invasive through a combination of molecular and behavioural alterations. Characteristic markers of EMT include expression shifts from E-cadherin to N-cadherin, increased activation of proteins like β-catenin and increased expression of fibronectin and vimentin (Huang et al. 2022).

CAFs also play a key role in PDAC progression. PSCs are resident in the pancreatic stroma and a major source of CAFs. PSCs in a quiescent state are characterised by the presence of vitamin A-containing lipid droplets before losing this phenotype and acquiring an activated myofibroblast morphology following prolonged culture and spreading on tissue culture plastic and in response to disrupted stromal homeostasis during PDAC (Menezes et al. 2022).

**Fig. 1:**
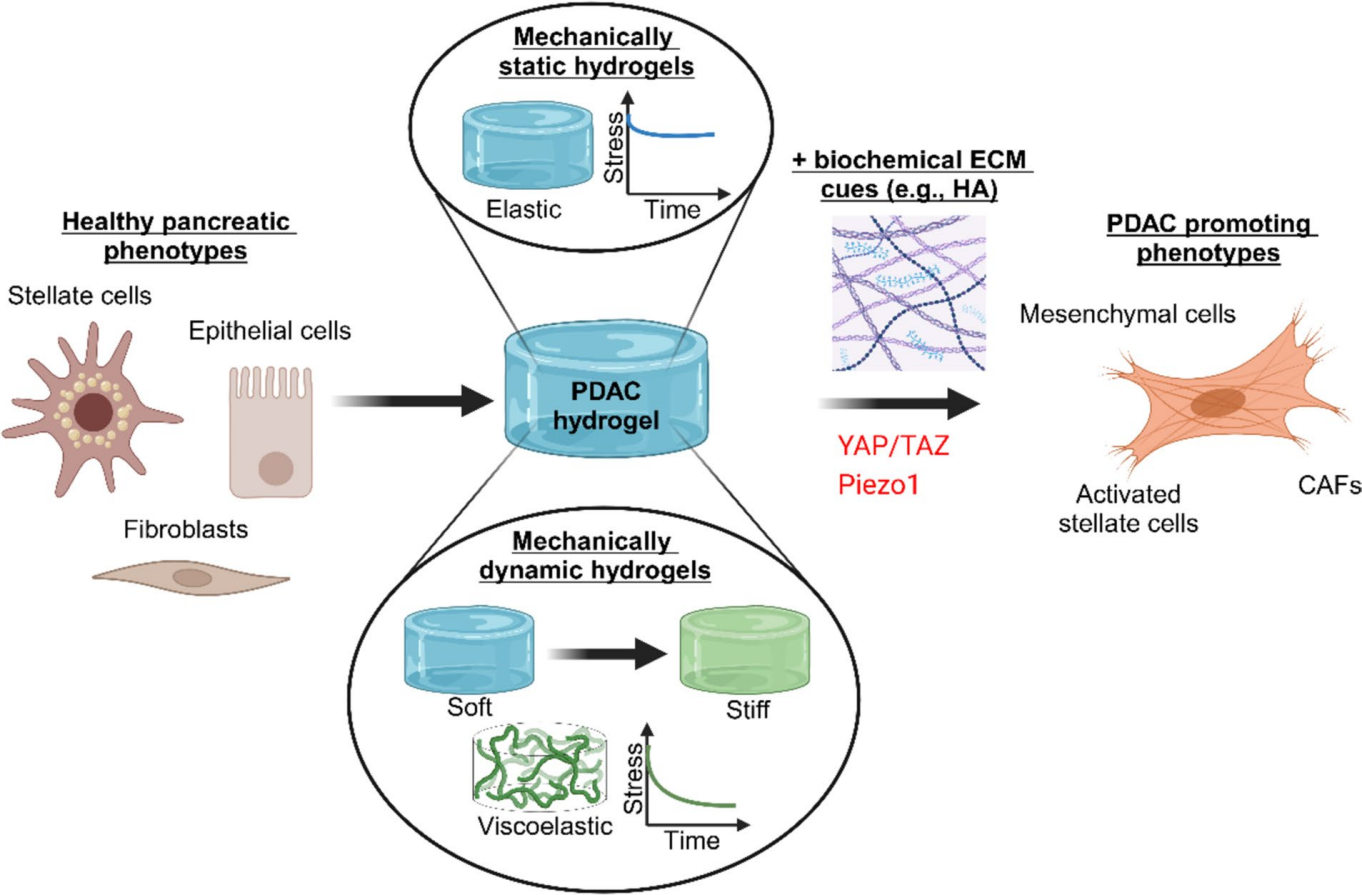
Schematic of hydrogel studies into the differentiation of healthy pancreatic cell types towards PDAC-promoting phenotypes by modelling PDAC mechanics using hydrogels. Hydrogel studies focused on static and dynamic systems with synergistic roles of biochemical ECM cues and identified key mechanobiological signalling players in YAP/TAZ and Piezo1 for mediating cell responses

Here, we discuss the role of static and dynamic mechanical cues, as well as synergistic effects with ECM components, on cell differentiation in PDAC. A figure summarising the role of mechanics on cell differentiation during PDAC using hydrogel models is shown in Fig. 1.

#### Static mechanics

Since increased stiffness is known to occur during PDAC progression, the elastic response of hydrogels has been used to understand pathological cell responses during the mechanosensing of biomechanical properties of the ECM. Many studies have designed hydrogels with carefully controlled elastic properties using numerous characterisation methods to confirm this; it should be considered that different mechanical characterisation approaches can yield different moduli.

EMT has been extensively studied in this context; for instance, Liu et al. showed that primary human PDAC cells encapsulated in 3D self-assembled peptide gels underwent EMT in stiffer environments; this response was mediated by YAP/TAZ mechanosensing and occurred in gels between 1 and 10 kPa that were characterised by rheometry (Liu et al. 2024). Ermis et al. developed HA methacryloyly (HAMA)-gelatin methacryloyl (GelMA) hydrogels mimicking healthy tissue and PDAC desmoplasia rigidities between 3, 15 and 36 kPa stiffness, which were characterised by compressive mechanical testing and rheometry. In this study, it was shown that stiffer mechanics induced mechanosensitive EMT of ASPC-1 cells, particularly when co-cultured with CAFs, through increased YAP activation in 3D (Ermis et al. 2023). Rice et al. also demonstrated that increased stiffness of polyacrylamide gel matrices characterised by atomic force microscopy (AFM), which mimic healthy tissue, PanIN and PDAC fibrotic tissue rigidities (1, 4, 24 kPa), promoted EMT of various PDAC epithelial cell lines (BxPC-3, Suit2-007 and AsPC-1) in a YAP/TAZ-mediated manner in 2D (Rice et al. 2017).

Budde et al. showed that primary PSCs become activated on stiffer 2D polyacrylamide substrates (between 0.75, 5 and 13.5 kPa, characterised by AFM), with increased durotaxis, which was found to be dependent on the activity of various force-sensitive ion channels, particularly Piezo1 (Budde 2024). Lachowski et al. also used polyacrylamide gels of 1 and 25 kPa stiffness that were developed based on previous literature using AFM to show that primary human PSC activation in 2D is mechanosensitive, with stiffer substrates found to activate PSCs to a myofibroblast-like phenotype (Lachowski et al. 2017).

#### Dynamic mechanics

Generally, hydrogel models of PDAC have been studied as mechanically static materials, characterised only by their elastic modulus or stiffness. However, both the ECM and many hydrogel materials exhibit viscoelasticity, which involves some viscous, stress-relaxing behaviour in which mechanics change in a time-dependent manner. Many studies disregard this dissipative contribution and do not attempt to either control or alter it. In this respect, hydrogels are considered elastic, or in any case, slow relaxing, and the observed cell responses are ascribed to changes in substrate stiffness alone. However, viscoelasticity is extremely relevant in biomedical applications since almost all bodily tissues display stress relaxation behaviour from the contributions of both elastic and viscous properties (Chaudhuri et al. 2020). Intriguingly, the mechanosensitive cell mechanism of responding to ECM viscous properties is conserved with the response to elasticity, which operates via the molecular clutch model that transduces mechanical stimuli into biochemical signals to regulate cell response (Bennett et al. 2018; Cantini et al. 2020). The balance between elastic and viscous ECM components is critical in dictating cell mechanosensitive responses by altering stress relaxation behaviour to regulate molecular clutch binding timescales; this has been modelled to predict cell spreading behaviour (Gong et al. 2018).

Biomechanics in PDAC has been shown to change over time and display changes in both elasticity and viscosity at the PDAC stage and earlier during pancreatitis compared to healthy pancreatic tissue (Rubiano et al. 2018). Based on this knowledge, it is critical that the role of viscous mechanics within the ECM is considered in regulating pathological cell responses during PDAC progression, particularly at early stages of ADM, pancreatitis and PanIN development, which may reveal novel early onset diagnostic and therapeutic targets.

Recent studies have made efforts to recapitulate dynamic alterations in tissue mechanics with carefully designed hydrogel matrices using numerous fabrication strategies to allow studies into time-dependant cell mechanosensing and behavioural response. These strategies can include encouraging physical entanglement of polymer chains by either incorporating high molecular weight viscous polymers or altering polymer:crosslinking ratios and introducing more dynamic crosslinking chemistries (Ma et al. 2021; Chaudhuri 2017). Some of these strategies have been employed in recent studies to explore the role of altered viscous properties in PDAC progression.

Nguyen et al. developed a highly flexible dynamic hydrogel using an adapted gelatin-norbornene system that can independently increase elastic and viscous properties as measured by rheometry. In this study, dynamic increases in elasticity from 2 to 6 kPa, rather than viscosity, enhanced EMT of COLO-357 cells in 3D (Nguyen and Lin 2024). Lin et al. engineered 3D hydrogels with tuneable viscoelastic properties, as measured by rheometry using poly (oligo (ethylene glycol) acrylate-s-hydroxyethyl acrylate-s-acrylamidophenylboronic acid (poly (OEGA-s-HEAA-s-APBA or PEHA) and poly (HEAA)-dopamine (PHD))–based materials that were conjugated with norbornene functional groups to yield PEH_NB_ and PEH_NB_A as thiol-norbornene photocrosslinkable macromers. With these hydrogels, they demonstrated that by mimicking the stiffness of PDAC tissue at ~ 6 kPa, an increase in viscous properties alone significantly enhanced EMT of COLO-357 and PANC-1 cells; when co-cultured with CAFs, this further led to upregulation of ECM remodelling genes and downregulation of tumour-suppressor genes (Lin et al. 2023).

Another method of dynamically altering hydrogel mechanics is to stiffen the environment in a triggered manner to recapitulate real-time changes in mechanics that coincide with those seen during disease progression. This has been shown to be effective in probing short- and long-term cellular responses to dynamic mechanics through a combination of modulating adhesion, traction and intracellular tension (Guvendiren and Burdick 2012). Given the dynamic nature of PDAC progression as the tissue develops over time from a healthy state to PDAC, modelling these mechanical transitions using hydrogels could be an effective and more representative approach for characterising cell response.

Chang et al. developed a cell-laden dually modified gelatin macromer–gelatin-norbornene-carbohydrazide (GelNB-CH) hydrogel system for 2D and 3D culture that is capable of being dynamically stiffened while accumulating HA, which is a key glycosaminoglycan (GAG) that is increasingly deposited by stromal cells during tumour progression. Dynamic stiffening between 2 and 30 kPa with HA-based macromers was measured via rheometry and triggered higher CD44 expression in PANC-1 spheroids, which correlated with increased proliferation and a more mesenchymal-like phenotype; CAFs exposed to dynamic stiffening in this system displayed a more mesenchymal, invasive morphology (Chang et al. 2021).

#### Synergy with ECM cues

Alongside altered biomechanics, the biochemical composition of the PDAC ECM is significantly altered during tumour progression, and this plays a critical role in regulating pathological cell behaviour. Recent works have highlighted how biochemical ECM cues and mechanosensing are critical for synergistically regulating cell response in PDAC.

Liu et al. developed a dynamic gelatin-HA hybrid hydrogel system by integrating modular thiol-norbornene photopolymerization and enzyme-triggered on-demand matrix stiffening, which was characterised by rheometry. This work showed that either dynamically stiffened gels between 1 and 8 kPa or HA-containing matrices individually inhibited the growth of COLO-357 cells; synergistically, however, these collective components were able to drive phenotypic changes associated with PDAC progression, including increased migration, invasion and EMT in 3D (Liu et al. 2018).

### Chemoresistance

**Fig. 2:**
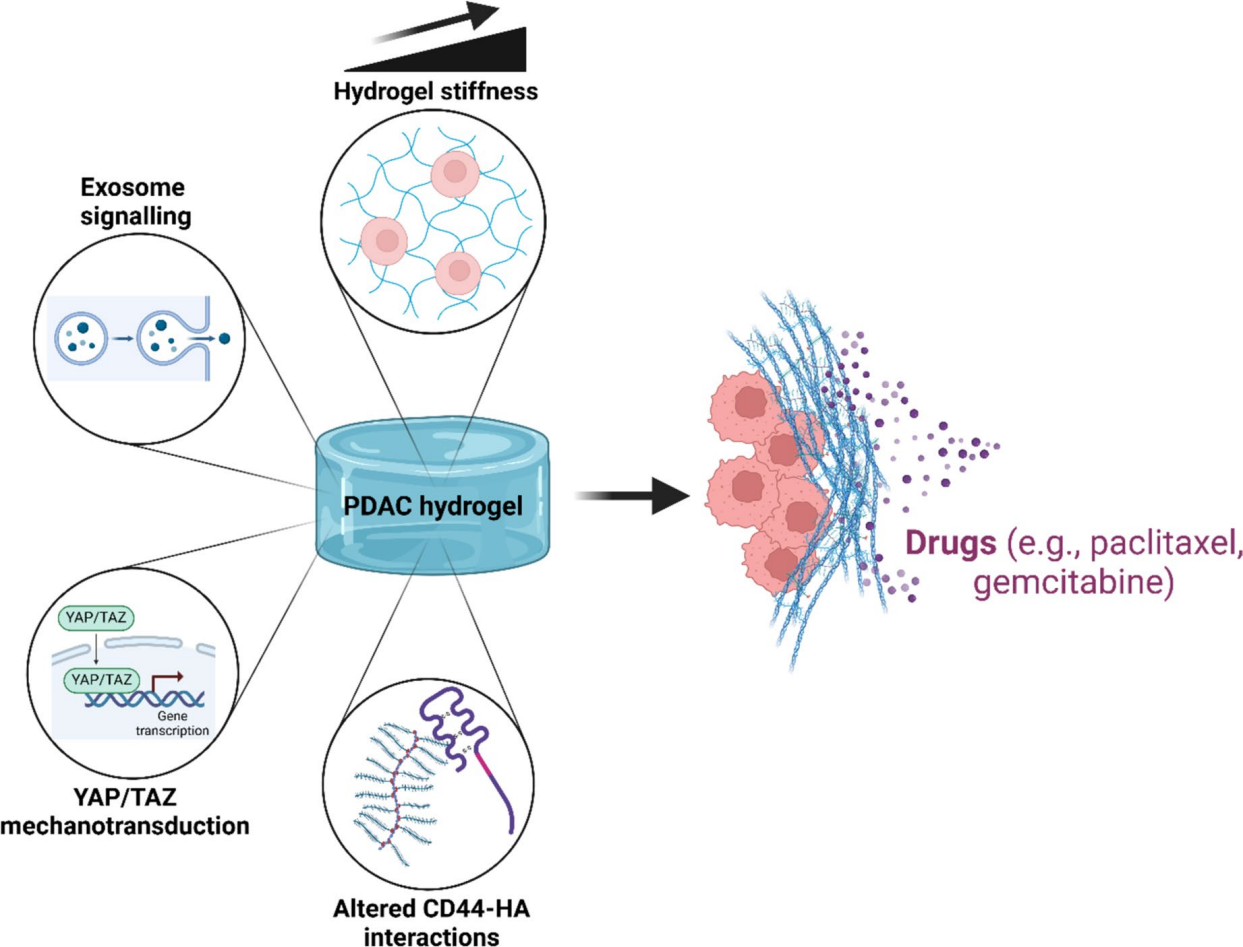
Schematic of hydrogel studies into mechanosensitive chemoresistance during PDAC with a focus on modulating matrix stiffness, YAP/TAZ mechanotransduction, altered CD44-HA interactions and exosome-mediated signalling

As cells abnormally differentiate into phenotypes associated with PDAC, enhanced chemoresistance often accompanies these phenomena, which coincides with the extreme fibrotic remodelling of the ECM that has a characteristic high stiffness and dense stroma (Feig et al. 2012; Hosein et al. 2020). Several ECM components secreted by both PDAC and stromal cells have been found to correlate with poor patient survival (Tian et al. 2019). In PDAC, these matrix properties and the associated high interstitial fluid pressures act to limit drug efficacy (DuFort et al. 2016). Encouragingly, co-administration of chemotherapies and ECM-depleting factors can significantly improve survival rates in mice (Olive et al. 2009; Provenzano et al. 2012). However, these therapies based on animal models have yet to show efficacy in human PDAC trials (Hakim et al. 2019; Catenacci et al. 2015). The development of effective human-derived ECM models is therefore critical in understanding chemoresistance in PDAC. A summary of recent approaches to studying mechanosensitive chemoresistance in PDAC to hydrogel models is shown in Fig. 2.

LeSavage et al. showed that increased ECM stiffness drives PDAC chemoresistance in 3D using patient-derived epithelial organoids grown in HA and elastic-like protein (ELP) hydrogels, termed HELP, which ranged between 0.2 and 3 kPa following mechanical characterisation by rheometry. Matrix-induced chemoresistance to gemcitabine was observed within a stiff environment due to the increased expression of drug efflux transporters, mediated by CD44 receptor interactions with hyaluronan, which was reversible following transfer to softer matrices (LeSavage et al. 2024).

Exosomes are intriguing, secreted molecules from cells that may have the potential for treatment and diagnosis of PDAC (Papadakos et al. 2022). Exosomes are nanoparticles typically sized between 30 and 150 nm, which are involved in a variety of signalling cascades that are determined by the interconnection between exosome surface proteins and receptors on recipient cells (Kalluri and LeBleu 2020; Gurung et al. 2021). Diagnostically, it has been shown that exosomes contain specific molecules, such as Glypican-1 and CA19-9, from circulating tumour cells that can be used for diagnostic and prognostic purposes for patients with resectable tumours (Buscail et al. 2019a, b). Exosomes secreted in pancreatic juice are capable of distinguishing between PDAC and chronic pancreatitis (Nakamura et al. 2019). From a treatment perspective, most studies have shown positive effects in terms of reduction of drug resistance, the utilisation of exosomes as drug-carries and therapeutic modification of the immune TME (Papadakos et al. 2022).

Exosomes from cells exposed to PDAC-mimetic conditions have been found to contain critical signalling molecules that transfer chemoresistance to recipient PDAC cells following secretion from drug-resistant CAFs (Richards et al. 2017). In terms of mechanosensitive exosome release, Xiao et al. used 3D co-cultures of primary PDAC organoids and CAFs, isolated from mice models in Matrigel/collagen-I gels to demonstrate that higher stiffness environments were established by cellular remodelling from 1 to 3 kPa, as measured by rheometry, which mimicked desmoplasia mechanics. These mechanical changes enhanced the chemoresistance of PDAC organoids through exosome-mediated signalling by CAFs that were mechanically hyperactivated in a YAP-mediated manner (Xiao et al. 2022).

In the context of EMT, Liu et al. showed that primary human PDAC cells encapsulated in 3D self-assembled peptide gels underwent YAP/TAZ mechanosensing-dependent EMT in stiffer environments, between 1 and 10 kPa characterised by rheometry, resulting in increased resistance to chemotherapeutic drugs gemcitabine and nab-paclitaxel (Liu et al. 2024). Rice et al. demonstrated that increasing the stiffness of 2D polyacrylamide gel matrices to mimic healthy, early lesion and PDAC tissue rigidities (1, 4 and 24 kPa, measured using AFM characterisation) promoted YAP/TAZ-mediated EMT of various PDAC epithelial cell lines (BxPC-3, Suit2-007 and AsPC-1) in a stiffness-dependent manner and increased chemoresistance to paclitaxel, but not gemcitabine, suggesting that specific therapeutic approaches may be required to target the mechanosensitive response in PDAC (Rice et al. 2017).

### Metastasis

Once the primary tumour is established as a densely fibrotic, chemoresistant entity, the dissemination of malignant cells to distant sites and the formation of metastatic nodules is a key step in PDAC progression and aggressiveness. EMT is a critical event during PDAC metastasis, which involves migration and invasion from the primary tumour into the ECM. Using protrusive cellular features, such as invadopodia, differentiated mesenchymal cells degrade and traverse through the stroma before extravasation into the bloodstream or lymphatics, where they circulate and potentially form distant metastases elsewhere in the body (Joshi et al. 2023). The most common site of metastasis in PDAC is the liver, which comprises over 60% of patients; this is followed by the lungs at around 30%, and bone accounts for around 10% (Ayres Pereira and Chio 2019). There are several models that are used in the study of metastasis, which each have specific advantages and disadvantages; these have been reviewed extensively by Ayres Pereira and Chio. To summarise, 2D models of cells seeded on substrates, such as hydrogels, have advantages in terms of their cost-effectiveness and ease of setup; they also are effective to study certain processes in metastasis such as EMT, migration and ECM interactions; however, these models suffer from lacking the complexity and dimensionality of human tissue architectures. 3D models are much closer to mimicking the complexity and dimensionality of human tissue, including oxygen and nutrient gradients, and permit co-cultures with 3D interactions; however, setting up 3D systems is more time-consuming, expensive and more challenging to study migration. Zebrafish models are effective in vivo systems for studying PDAC due to highly conserved developmental mechanisms between zebrafish and mammals, easy monitoring of PDAC progression and high cell survival and metastatic capability within this system; they are effective models for studying invasion, survival in circulation and extravasation; however, studies in early embryos do not represent a fully immune-competent host. Chick embryo models have been used to study PDAC metastasis and are advantageous due to their cost effectiveness, fast and simple imaging of migration and metastasis, and no surgical requirements; they are used for similar types of studies as zebrafish models but suffer from reproducibility issues and inconsistencies in tumour grafting. Genetically engineered mouse models can recapitulate the histopathological features of human PDAC and have an intact, immune-competent TME; dissemination can be studied via lineage tracing models (such as fluorescently labelled cells), which are important to investigate invasion and survival in circulation; these models are however costly, difficult to monitor tumours and tumour development is generally delayed. Allograft in vivo models have also been used since they have an intact immune system to account for its involvement in metastasis, accurate recapitulation of the PDAC TME and allows for the rapid and consistent establishment of tumours and metastasis; however, a limitation of the approach is that donor cells are not of human origin. Xenograft in vivo models offer an advantage over allograft systems since donor cells are of human origin; they also have relatively low cost and rapid tumour growth; however, they require an immune-deficient host; the tumour stroma is of mouse origin, and not all models are capable of developing metastasis. Applications of both allograft and xenograft models include invasion, circulation and extravasation (Ayres Pereira and Chio 2019). Due to the significant differences between using specific metastasis models, it is important to consider the advantages and disadvantages of each and ascertain which type of model is most appropriate to study a particular cell response involved in metastasis.

**Fig. 3:**
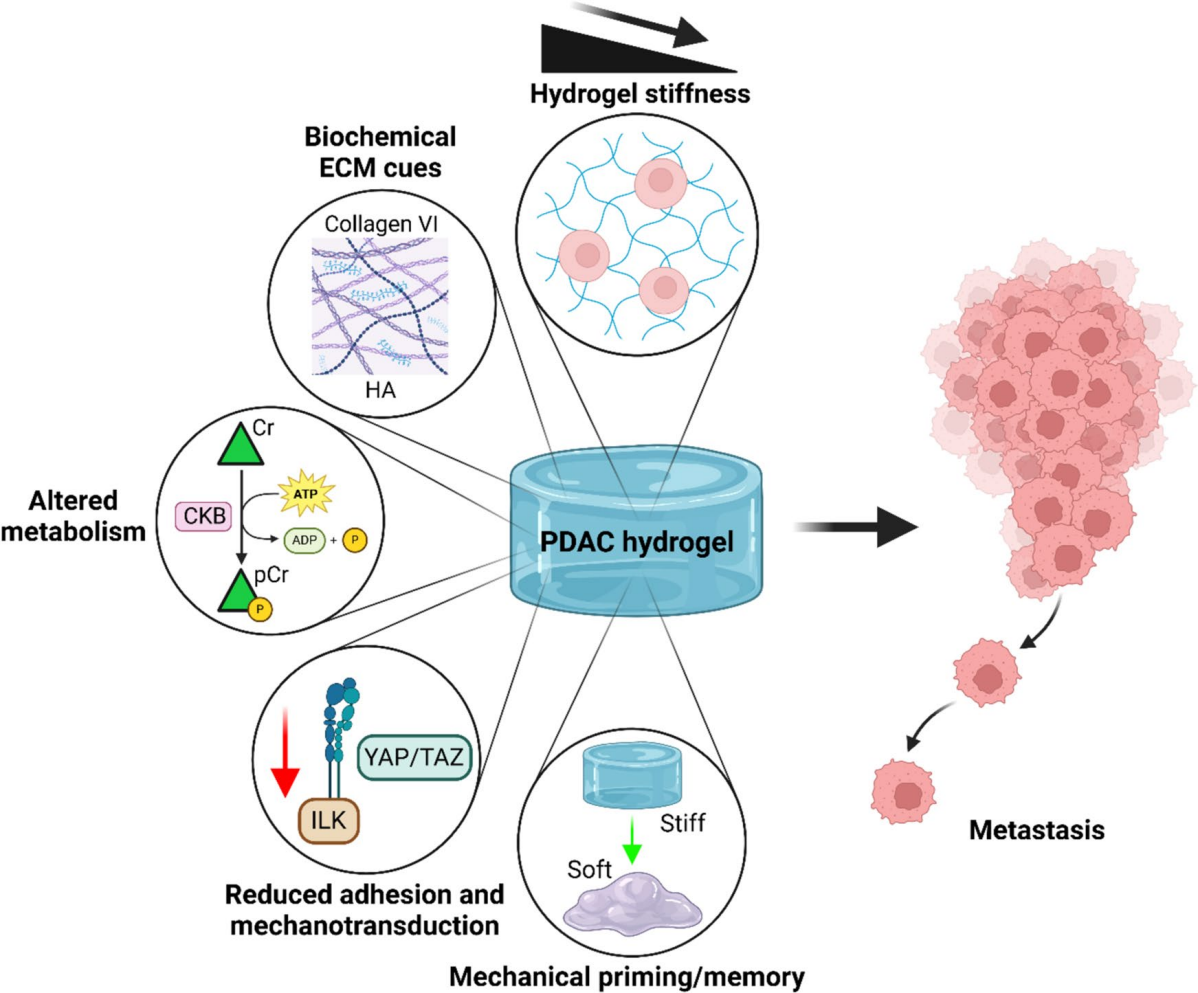
Schematic of hydrogel studies into mechanosensitive metastasis during PDAC with a focus on modulating matrix stiffness, YAP/TAZ mechanotransduction and integrin signalling, mechanical priming/memory of cells, specific biochemical ECM cues and altered metabolism

The ECM is a key component of the metastatic niche, and emerging evidence suggests that cancer cell interactions with the biochemical and biophysical components of the ECM play key roles in the establishment of metastatic tumours at all stages of the metastatic cascade (Drew and Machesky 2021). A summary of recent approaches using hydrogel models to study the role of altered mechanosensing on metastasis is shown in Fig. 3.

Liu et al. showed that PDAC cell growth was inhibited in 3D using either dynamically stiffened gels between 1 and 8 kPa (characterised by rheometry) or HA-containing matrices. However, when combined, these components were able to drive phenotypic changes in COLO-357 cells that are associated with PDAC progression, including increased migration and invasion, which are key metastatic traits (Liu et al. 2018). Nguyen et al. showed that COLO-357 cells displayed a more invasive morphology in 3D gelatin-norbornene gels that dynamically stiffen from 2 to 6 kPa (as measured by rheometry), indicating an enhanced metastatic tendency in a stiffer mechanical environment (Nguyen and Lin 2024). Lin et al. showed using engineered 3D PEH_NB_ and PEH_NB_A hydrogels with tuneable viscoelastic properties at a stiffness of ~ 6 kPa, as measured by rheometry, that COLO-357 and PANC-1 cells displayed significantly downregulated tumour metastasis suppressor genes in more viscous environments (Lin et al. 2023).

Mechanical priming involves culturing cells in one mechanical environment and transferring them to another, such as stiff to soft; this is a common approach to studying how mechanical memory influences the ability of invading cancer cells to ‘remember’ the stiff mechanical properties of the primary TME even while invading a healthy softer region (Dudaryeva et al. 2023). Additionally, engineered models of cellular confinement within their microenvironment, which is a key feature during cancer pathology, have been used to highlight its key role in 3D for regulating cellular mechanosensation and regulating gene expression changes during metastasis (Paul et al. 2016). Atkins et al. developed soft and stiff 3D polyacrylamide microgels that mimicked healthy and PDAC pancreatic tissue mechanics (1 kPa and 10 kPa as measured by rheometry) to study the influence of mechanical priming and cellular confinement on PDAC metastatic potential. They showed that PDAC cells (cell line harbouring a mutated KRAS^G12D^) primed in stiff environments displayed significantly elevated marker expression of metastatic gene RAC3 than those in soft counterparts (Atkins et al. 2024).

Specific metabolic pathways have been associated with facilitating cancer progression. Oncogenic mutations impart metabolic flexibility to tumours, which allows aerobic glycolysis and confers a survival advantage despite the hostile environment (Lyssiotis and Kimmelman 2017). Tumour cells adjust their metabolism, which enables their survival despite limited nutrients, through tactics including scavenging for extracellular protein (Kamphorst et al. 2015) and rewiring their glucose metabolism (Ying et al. 2012). An interesting factor that has been associated with the cytoskeletal activity is cytoplasmic creatine kinase B-type (CKB), which can facilitate cell motility by providing ATP recycling locally at sites of actin remodelling (Kuiper et al. 2009). Increasing evidence also suggests that the phosphocreatine-creatine kinase (pCr-CK) system is important in malignancies (Fenouille et al. 2017; Kurmi et al. 2018). Papalazarou et al. revealed mechanosensitive insights into the role of CKB-mediated pCr-CK metabolism in PDAC using 2D polyacrylamide hydrogels with stiffness values of 0.7 kPa, 7 kPa and 38 kPa, as measured by AFM. Their findings suggest that in stiffer environments, CKB plays a critical role following YAP-mediated transcriptional regulation to drive phosphocreatine synthesis, cancer cell invasion, chemotaxis and metastasis of primary murine PDAC cells and PANC-1 cells (Papalazarou et al. 2020).

Papalazarou et al. also showed, using 2D polyacrylamide hydrogels with stiffness values of 0.7 kPa, 7 kPa and 38 kPa, characterised by AFM, that collagen VI is specifically upregulated in primary murine and PANC-1 pancreatic cancer cells on soft substrates due to a lack of integrin engagement. Indeed, the expression of collagen VI coincided with higher focal adhesion turnover and inversely correlated with the activity of YAP and ILK. Increased collagen VI supports migration in vitro and metastasis formation in vivo, and metastatic nodules formed by pancreatic cancer cells lacking *Col6a1* display stromal cell-derived collagen VI deposition, suggesting that cancer and stromal cells form key interactions within the metastatic niche and that collagen VI is an essential component of the niche (Papalazarou et al. 2022).

## Outlook

Hydrogels, due to their high mechanical tuneability, provide effective tools to study the mechanosensitive processes that regulate pathological cell responses in PDAC. Modelling the increased stiffness associated with PDAC desmoplasia has been the predominant mechanobiological focus of recent studies. High stiffness cell mechanosensing coincides with the activation of key signalling molecules, such as the mechanical rheostat YAP/TAZ, which facilitate cell responses that drive PDAC progression; these include differentiation to pathogenic phenotypes, increased chemoresistance and increased metastatic potential. A summarising table of each study discussed in this review is shown in Table 1; this covers key aspects within each study, such as the cells and materials used, type of cell response, with any associated signalling and mechanical properties studied.

**Table 1:** Summary of recent hydrogel studies into mechanosensing during PDAC by investigating differentiation, chemoresistance and metastasis

Cell response	Cell type	Stromal cell co-culture inclusion	Material used	Mechanical properties	Mechanical characterisation technique	Influence of mechanosensing	Associated mechanosensitive signalling	Dimensionality	References
EMT and chemoresistance	Primary human PDAC cells		Self-assembled peptide	1, 10 kPa	Rheometry	Increased stiffness correlated with an EMT phenotype and chemoresistance to gemcitabine and nab-paclitaxel	EMT and chemoresistance correlated with increased YAP/TAZ activation	3D	Liu et al. 2024)
EMT	ASPC-1	Primary CAFs	HA-GelMA	3, 15, 36 kPa	Compressive mechanical testing and rheometry	Increased stiffness correlated with an EMT phenotype	EMT correlated with increased YAP activation	3D	Ermis et al. 2023)
EMT and chemoresistance	BxPC-3, Suit2-007, AsPC-1		Polyacrylamide	1, 4, 24 kPa	AFM	Increased stiffness correlated with an EMT phenotype and increased chemoresistance to paclitaxel but not gemcitabine	EMT correlated with increased YAP/TAZ activation	2D	Rice et al. 2017)
EMT and metastasis	COLO-357		Gelatin-norbornene	2, 6 kPa	Rheometry	Dynamic increases in elasticity, rather than viscosity, correlated with an EMT phenotype and more invasive behaviour		3D	Nguyen and Lin 2024)
EMT and metastasis	COLO-357, PANC-1	Immortalised human CAFs	PEH_NB_, PEH_NB_A	6 kPa	Rheometry	At a constant stiffness, more viscous matrices enhance EMT and CAF-induced downregulation of tumour- and metastasis-suppressor genes	ROCK inhibition was found to partially inhibit CAF spreading behaviour, which may contribute towards their pathological behaviour within this system	3D	Lin et al. 2023)
EMT and metastasis	COLO-357		Gelatin-norbornene-HA	1, 8 kPa	Rheometry	Dynamic stiffening or HA-containing matrices alone inhibited cell growth. Synergistically, these factors enhanced EMT, migration and invasion		3D	Liu et al. 2018)
EMT, CAF activation	PANC-1, immortalised human CAFs		GelNB-CH-HA	2, 30 kPa	Rheometry	Dynamic stiffening with HA-macromers m induced an increased mesenchyme-like morphology of PANC-1 cells and a more invasive CAF morphology	Increased CD44 expression correlated with more cancer-associated cell phenotypes	2D, 3D	Chang et al. 2021)
PSC activation	Primary PSCs		Polyacrylamide	0.75, 5, 13.5 kPa	AFM	Increased stiffness correlated with PSC activation and increased durotaxis	PSC activation and durotaxis correlate with increased Piezo1 activity	2D	(Budde 2024)
PSC activation	Primary human PSCs		Polyacrylamide	1, 25 kPa	AFM	Increased stiffness correlated with PSC activation to a myofibroblast phenotype		2D	Lachowski et al. 2017)
Chemoresistance	Primary murine PDAC organoids	Primary murine CAFs	Matrigel/collagen-I	1, 3 kPa	Rheometry	Increased ECM stiffening by cellular remodelling enhanced chemoresistance of PDAC organoids through exosome-mediated signalling by CAFs that were mechanically hyperactivated	Increased YAP correlated with CAF activation and exosome secretion that conferred chemotherapeutic drug resistance to PDAC organoids	3D	Xiao et al. 2022)
Chemoresistance	Patient-derived epithelial organoids		HELP	~ 0.1, ~ 1, ~ 3 kPa	Rheometry	Higher stiffness enhanced chemoresistance to gemcitabine by increased expression of drug efflux transporters, which was reversible following transfer to softer matrices	Drug efflux was mediated by CD44 interactions with hyaluronan	3D	LeSavage et al. 2024)
Metastasis	PDAC cell line (with mutate KRAS^G12D^)		Polyacrylamide	1, 10 kPa	Rheometry	Cells mechanically primed in a stiff environment display significantly elevated marker expression of metastatic gene RAC3 than those in soft counterparts		3D	Atkins et al. 2024)
Metastasis	Primary murine PDAC and PANC-1 cells		Polyacrylamide	0.7, 7, 38 kPa	AFM	Stiffer environments promote cell invasion, chemotaxis and metastasis	CKB plays a critical role following YAP-mediated transcriptional regulation to drive phosphocreatine synthesis	2D	Papalazarou et al. 2020)
Metastasis	Primary murine PDAC and PANC-1 cells		Polyacrylamide	0.7, 7, 38 kPa	AFM	Softer environments promote increased migration in vitro and metastasis formation in vivo	Collagen VI is a central player in driving metastasis during soft ECM mechanosensing and coincides with a lack of integrin engagement and reduced activity of ILK and YAP	2D	Papalazarou et al. 2022)

Studies were performed using various cell types, co-culture systems, different hydrogel materials, 2D and 3D environments, varied mechanical properties and characterisation methods and associated mechanosensitive signalling processes

Despite these common observations, there are still nuances associated with mechanosensing during PDAC to be further investigated; for instance, Papalazarou et al. showed in a series of studies that mechanosensing can facilitate metastasis differently in both soft and stiff environments. In one study, a stiffer environment was found to enhance cellular tension and YAP-mediated metastasis; this was specifically associated with altered metabolic activity (Papalazarou et al. 2020); however, a second study highlighted that softer environments support metastasis through lowering intracellular tension and the secretion of collagen VI with downregulation of integrins, ILK and YAP (Papalazarou et al. 2022). Clearly, there are various adaptive mechanisms that cells employ to facilitate processes associated with PDAC in different mechanical environments, which are known to exist within the tissue (Rice et al. 2017). These models can hence facilitate the identification of potential mechanosensitive therapeutic targets to disrupt abnormal cell events.

Another drawback of current studies is the fact that there is no standardised method for the mechanical characterisation of hydrogel models. Because of this, different mechanical moduli are derived from various sources, which introduces inherent differences between studies based on how the mechanical properties are derived using different instrumental apparatus. To overcome this, a standardised approach within the field would be ideal for more accurate comparisons and effective reproducibility between studies.

The role of viscoelasticity is intriguing within the context of PDAC; seemingly, there are nuances associated with its role in regulating pathological cell responses, such as EMT and metastasis. In the two studies discussed in this review, we already see conflicting responses despite using the same cell type (COLO-357 cells); Nguyen et al. showed using a gelatin-norbornene gel system that increased elasticity between 2 and 6 kPa, rather than viscosity, was the driving factor in enhancing EMT and invasive cell behaviour (Nguyen and Lin 2024), whereas Lin et al. showed that 6 kPa PEH_NB_ and PEH_NB_A hydrogels with more viscous nature enhanced EMT and metastatic potential (Lin et al. 2023). These differences could also be due to different material systems, which retain specific bioactivities that the cells also respond to, and therefore, further studies are required to elucidate the role of viscoelasticity in PDAC more clearly.

The dimensionality of currently used PDAC hydrogel models is a feature that varies between studies, with some focusing on 2D and others more on 3D. 2D systems, such as polyacrylamide, generally offer simpler and more reproducible hydrogel platforms to work with for understanding fundamental cell mechanosensitive processes over their 3D counterparts. However, Baker et al. highlighted the influence of dimensionality on mechanosensitive cell response and outlined clear differences between 2 and 3D systems in the context of cancer (Baker and Chen 2012). Since PDAC occurs in a 3D environment, it is crucial that studies progress towards developing more 3D systems to accurately recapitulate the dimensionality that cells encounter in physiological and pathological conditions.

Given the importance of pancreatitis and PanIN as early predictive markers of PDAC onset, it is critical that we develop effective hydrogel models that recapitulate the biophysics of these microenvironments to potentially reveal novel diagnostic and therapeutic markers linked to mechanobiology. Indeed, previous studies have performed measurements on these stages of disease progression and shown distinct differences in their viscoelastic properties, which could be critical in regulating abnormal cell responses (Rice et al. 2017; Rubiano et al. 2018). Previously, difficulties in modelling the viscoelastic mechanics of pancreatitis and PanIN tissue arose, likely due to a lack of knowledge and methodologies/materials to fabricate hydrogels with controlled viscoelasticity; however, in more recent works, there is a much greater repository of viscoelastic hydrogel engineering strategies that should help overcome previous challenges (Ma et al. 2021). Going forward, it would be highly informative to develop hydrogels with controlled viscoelastic mechanics to provide novel insights into mechanosensitive diagnostic and therapeutic targets during pancreatitis and PanIN stages of PDAC progression.

Another area that warrants further investigation in PDAC mechanobiology is metabolism. Despite the key roles of altered metabolism and biophysics in PDAC progression, very little has been studied in the context of how these are interconnected. Papalazarou et al. revealed mechanosensitive insights into the role of CKB-mediated pCr-CK metabolism in PDAC by showing that CKB plays a critical role following YAP-mediated transcriptional regulation to drive phosphocreatine synthesis, cancer cell invasion, chemotaxis and metastasis (Papalazarou et al. 2020). Another key area that has been implicated in metabolic dysregulation during PDAC progression is autophagy; this describes the breakdown and recycling of intracellular components, particularly during periods of stress or starvation (Piffoux et al. 2021). Hupfer et al. showed a mechanical link in PDAC between ECM stiffness, metabolism and autophagy by revealing that stiffer environments led to PDAC tumour growth through enhanced autophagy responses that were dependent upon AMP-activated protein kinase (AMPK) downregulation of lipid biosynthesis by PSCs (Hupfer et al. 2021). Further studies in this area are critical for advancing our knowledge of linking altered cell mechanosensing during changes in PDAC tissue mechanics to dysregulated cellular metabolism; this could provide novel clinical insights for diagnostic and therapeutic targeting.

Exosomes are another key signalling molecule heavily implicated in PDAC, for instance, by transferring chemoresistance between cells. The role of mechanobiology in the exosome secretory response is poorly understood, and thus, more studies are needed in this area to reveal their diagnostic and therapeutic potential.

In the future, more physiologically representative hydrogel systems may lead to further insights. Below et al. recently created a synthetic 3D matrix that recapitulated both the biochemical and mechanical features of the PDAC TME. By analysing mouse PDAC tissue and published human data, they defined a reference set of ECM components and used these to develop an eight-arm PEG-based hydrogel scaffold modified to include relevant ECM-mimetic and basement membrane–binding peptides. The stiffness of these hydrogels could also be modulated by altering the concentration of the crosslinking peptide. The authors found that these scaffolds mimicked the stiffness of the original tissue, enhanced organoid growth and resulted in upregulation of molecules associated with mechanosignalling. Importantly, they were also able to co-culture cancer cell organoids with fibroblasts, macrophages and myeloid cells, with viable cells maintained for up to 6 days. Embedded fibroblasts recapitulated the subtypes found in vivo, and the authors detected the secretion of cytokines and chemokines, suggesting interactions between tumour and stromal cells (Below et al. 2022).

Going forward, it will be essential to incorporate all relevant cell types in hydrogel systems to provide more insight into the PDAC TME, as well as consistency with mechanical characterisation approaches and more focus on 3D systems. It is also crucial that we build upon the mechanobiological knowledge gathered so far. Identification of novel mechanosensitive roles in PDAC, particularly at early stages of progression such as PanINs, will provide new insights into how cells perceive and respond to changes in mechanical stimuli, better informing PDAC researchers and clinicians for expansion of the therapeutic window and identification of novel diagnostic and therapeutic targets.

## Data Availability

No datasets were generated or analysed during the current study.

## References

[CR1] Andersson B, Appelgren B, Sjödin V, Ansari D, Nilsson J, Persson U et al (2013) Acute pancreatitis – costs for healthcare and loss of production. Scand J Gastroenterol 48(12):1459–1465. 10.3109/00365521.2013.843201 24131379 10.3109/00365521.2013.843201

[CR2] Asadishekari M, Mpoyi EN, Li Y, Eslami J, Walker M, Cantini M et al (2022) Three-dimensional tunable fibronectin-collagen platforms for control of cell adhesion and matrix deposition. Front Phys 10:806554. 10.3389/fphy.2022.806554

[CR3] Atkins DJ, Rosas JM, Månsson LK, Shahverdi N, Dey SS, Pitenis AA (2024) Survival-associated cellular response maintained in pancreatic ductal adenocarcinoma (PDAC) switched between soft and Stiff 3D microgel culture. ACS Biomater Sci Eng 10(4):2177–2187. 10.1021/acsbiomaterials.3c01079 38466617 10.1021/acsbiomaterials.3c01079 PMC11005012

[CR4] Ayres Pereira M, Chio IIC (2019) Metastasis in pancreatic ductal adenocarcinoma: current standing and methodologies. Genes 11(1):6. 10.3390/genes11010006 31861620 10.3390/genes11010006 PMC7016631

[CR5] Baker BM, Chen CS (2012) Deconstructing the third dimension – how 3D culture microenvironments alter cellular cues. J Cell Sci 125(13):3015–3024. 10.1242/jcs.079509 22797912 10.1242/jcs.079509 PMC3434846

[CR6] Below CR, Kelly J, Brown A, Humphries JD, Hutton C, Xu J et al (2022) A microenvironment-inspired synthetic three-dimensional model for pancreatic ductal adenocarcinoma organoids. Nat Mater 21(1):110–119. 10.1038/s41563-021-01085-1 34518665 10.1038/s41563-021-01085-1 PMC7612137

[CR7] Bennett M, Cantini M, Reboud J, Cooper JM, Roca-Cusachs P, Salmeron-Sanchez M (2018) Molecular clutch drives cell response to surface viscosity. Proc Natl Acad Sci USA 115(6):1192–1197. 10.1073/pnas.1710653115 29358406 10.1073/pnas.1710653115 PMC5819391

[CR8] Budde I, Schlichting A, Ing D, Schimmelpfennig S, Kuntze A, Fels B et al (2024) Piezo1-induced durotaxis of pancreatic stellate cells depends on TRPC1 and TRPV4 channels. bioRxiv: the preprint server for biology. 10.1101/2023.12.22.572956 10.1242/jcs.263846 PMC12136172 40019468

[CR9] Burdick JA, Prestwich GD (2011) Hyaluronic acid hydrogels for biomedical applications. Adv Mater 23(12):H41–H56. 10.1002/adma.201003963 21394792 10.1002/adma.201003963 PMC3730855

[CR10] Buscail E, Alix-Panabières C, Quincy P, Cauvin T, Chauvet A, Degrandi O et al (2019a) High clinical value of liquid biopsy to detect circulating tumor cells and tumor exosomes in pancreatic ductal adenocarcinoma patients eligible for up-front surgery. Cancers 11(11):1656. 10.3390/cancers11111656 31717747 10.3390/cancers11111656 PMC6895804

[CR11] Buscail E, Chauvet A, Quincy P, Degrandi O, Buscail C, Lamrissi I et al (2019b) CD63-GPC1-positive exosomes coupled with CA19-9 offer good diagnostic potential for resectable pancreatic ductal adenocarcinoma. Transl Oncol 12(11):1395–1403. 10.1016/j.tranon.2019.07.009 31400579 10.1016/j.tranon.2019.07.009 PMC6699195

[CR12] Caliari SR, Burdick JA (2016) A practical guide to hydrogels for cell culture. Nat Methods 13(5):405–414. 10.1038/nmeth.3839 27123816 10.1038/nmeth.3839 PMC5800304

[CR13] Cantini M, Donnelly H, Dalby MJ, Salmeron-Sanchez M (2020) The plot thickens: the emerging role of matrix viscosity in cell mechanotransduction. Adv Healthc Mater 9(8):1901259. 10.1002/adhm.201901259 10.1002/adhm.201901259 31815372

[CR14] Catenacci DV, Junttila MR, Karrison T, Bahary N, Horiba MN, Nattam SR et al (2015) Randomized phase Ib/II study of gemcitabine plus placebo or vismodegib, a hedgehog pathway inhibitor, in patients with metastatic pancreatic cancer. J Clin Oncol 33(36):4284–4292. 10.1200/jco.2015.62.8719 26527777 10.1200/JCO.2015.62.8719 PMC4678179

[CR15] Chang C-Y, Johnson HC, Babb O, Fishel ML, Lin C-C (2021) Biomimetic stiffening of cell-laden hydrogels via sequential thiol-ene and hydrazone click reactions. Acta Biomater 130:161–171. 10.1016/j.actbio.2021.05.054 34087443 10.1016/j.actbio.2021.05.054 PMC8316407

[CR16] Chaudhuri O (2017) Viscoelastic hydrogels for 3D cell culture. Biomater Sci 5(8):1480–1490. 10.1039/C7BM00261K 28584885 10.1039/c7bm00261k

[CR17] Chaudhuri O, Cooper-White J, Janmey PA, Mooney DJ, Shenoy VB (2020) Effects of extracellular matrix viscoelasticity on cellular behaviour. Nature 584(7822):535–546. 10.1038/s41586-020-2612-2 32848221 10.1038/s41586-020-2612-2 PMC7676152

[CR18] Chen J, Zou X (2019) Self-assemble peptide biomaterials and their biomedical applications. Bioact Mater 4:120–131. 10.1016/j.bioactmat.2019.01.002 31667440 10.1016/j.bioactmat.2019.01.002 PMC6812166

[CR19] Chu GC, Kimmelman AC, Hezel AF, DePinho RA (2007) Stromal biology of pancreatic cancer. J Cell Biochem 101(4):887–907. 10.1002/jcb.21209 17266048 10.1002/jcb.21209

[CR20] Drew J, Machesky LM (2021) The liver metastatic niche: modelling the extracellular matrix in metastasis. Dis Model Mech 14(4). 10.1242/dmm.048801 10.1242/dmm.048801 PMC8077555 33973625

[CR21] Dudaryeva OY, Bernhard S, Tibbitt MW, Labouesse C (2023) Implications of cellular mechanical memory in bioengineering. ACS Biomater Sci Eng 9(11):5985–5998. 10.1021/acsbiomaterials.3c01007 37797187 10.1021/acsbiomaterials.3c01007 PMC10646820

[CR22] DuFort CC, DelGiorno KE, Carlson MA, Osgood RJ, Zhao C, Huang Z et al (2016) Interstitial pressure in pancreatic ductal adenocarcinoma is dominated by a gel-fluid phase. Biophys J 110(9):2106–2119. 10.1016/j.bpj.2016.03.040 27166818 10.1016/j.bpj.2016.03.040 PMC4939548

[CR23] Duscher D, Maan ZN, Wong VW, Rennert RC, Januszyk M, Rodrigues M et al (2014) Mechanotransduction and fibrosis. J Biomech 47(9):1997–2005. 10.1016/j.jbiomech.2014.03.031 24709567 10.1016/j.jbiomech.2014.03.031 PMC4425300

[CR24] Ermis M, Falcone N, Roberto de Barros N, Mecwan M, Haghniaz R, Choroomi A et al (2023) Tunable hybrid hydrogels with multicellular spheroids for modeling desmoplastic pancreatic cancer. Bioact Mater 25:360–373. 10.1016/j.bioactmat.2023.02.005 36879666 10.1016/j.bioactmat.2023.02.005 PMC9984297

[CR25] Feig C, Gopinathan A, Neesse A, Chan DS, Cook N, Tuveson DA (2012) The pancreas cancer microenvironment. Clin Cancer Res 18(16):4266–4276. 10.1158/1078-0432.ccr-11-3114 22896693 10.1158/1078-0432.CCR-11-3114 PMC3442232

[CR26] Fenouille N, Bassil CF, Ben-Sahra I, Benajiba L, Alexe G, Ramos A et al (2017) The creatine kinase pathway is a metabolic vulnerability in EVI1-positive acute myeloid leukemia. Nat Med 23(3):301–313. 10.1038/nm.4283 28191887 10.1038/nm.4283 PMC5540325

[CR27] Gómez-Guillén MC, Giménez B, López-Caballero ME, Montero MP (2011) Functional and bioactive properties of collagen and gelatin from alternative sources: a review. Food Hydrocoll 25(8):1813–1827. 10.1016/j.foodhyd.2011.02.007

[CR28] Gong Z, Szczesny SE, Caliari SR, Charrier EE, Chaudhuri O, Cao X et al (2018) Matching material and cellular timescales maximizes cell spreading on viscoelastic substrates. Proc Natl Acad Sci U S A 115(12):E2686–E95. 10.1073/pnas.1716620115 29507238 10.1073/pnas.1716620115 PMC5866566

[CR29] Górska A, Mazur AJ (2022) Integrin-linked kinase (ILK): the known vs. the unknown and perspectives. Cell Mol Life Sci 79(2):100. 10.1007/s00018-021-04104-1 35089438 10.1007/s00018-021-04104-1 PMC8799556

[CR30] Gurung S, Perocheau D, Touramanidou L, Baruteau J (2021) The exosome journey: from biogenesis to uptake and intracellular signalling. Cell Commun Signal 19(1):47. 10.1186/s12964-021-00730-1 33892745 10.1186/s12964-021-00730-1 PMC8063428

[CR31] Guvendiren M, Burdick JA (2012) Stiffening hydrogels to probe short- and long-term cellular responses to dynamic mechanics. Nat Commun 3(1):792. 10.1038/ncomms1792 22531177 10.1038/ncomms1792

[CR32] Hakim N, Patel R, Devoe C, Saif MWJP (2019) Why HALO 301 failed and implications for treatment of pancreatic cancer. Pancreas 3(1):e1. 10.17140/POJ-3-e010 32030361 10.17140/POJ-3-e010 PMC7003617

[CR33] Halbrook CJ, Lyssiotis CA, di Magliano MP, Maitra A (2023) Pancreatic cancer: advances and challenges. Cell 186(8):1729–54. 10.1016/j.cell.2023.02.014 37059070 10.1016/j.cell.2023.02.014 PMC10182830

[CR34] Hellewell AL, Adams JCJB (2016) Insider trading: Extracellular matrix proteins and their non-canonical intracellular roles. Bioessays 38(1):77–88. 10.1002/bies.201500103 26735930 10.1002/bies.201500103

[CR35] Hosein AN, Brekken RA, Maitra A (2020) Pancreatic cancer stroma: an update on therapeutic targeting strategies. Nat Rev Gastroenterol Hepatol 17(8):487–505. 10.1038/s41575-020-0300-1 32393771 10.1038/s41575-020-0300-1 PMC8284850

[CR36] Huang Y, Hong W, Wei X (2022) The molecular mechanisms and therapeutic strategies of EMT in tumor progression and metastasis. J Hematol Oncol 15(1):129. 10.1186/s13045-022-01347-8 36076302 10.1186/s13045-022-01347-8 PMC9461252

[CR37] Hughes CS, Postovit LM, Lajoie GA (2010) Matrigel: a complex protein mixture required for optimal growth of cell culture. Proteomics 10(9):1886–1890. 10.1002/pmic.200900758 20162561 10.1002/pmic.200900758

[CR38] Humphrey JD, Dufresne ER, Schwartz MA (2014) Mechanotransduction and extracellular matrix homeostasis. Nat Rev Mol Cell Biol 15(12):802–812. 10.1038/nrm3896 25355505 10.1038/nrm3896 PMC4513363

[CR39] Hupfer A, Brichkina A, Koeniger A, Keber C, Denkert C, Pfefferle P et al (2021) Matrix stiffness drives stromal autophagy and promotes formation of a protumorigenic niche. Proc Natl Acad Sci USA 118(40):e2105367118. 10.1073/pnas.2105367118 34588305 10.1073/pnas.2105367118 PMC8501848

[CR40] Jiang H, Hegde S, Knolhoff BL, Zhu Y, Herndon JM, Meyer MA et al (2016) Targeting focal adhesion kinase renders pancreatic cancers responsive to checkpoint immunotherapy. Nat Med 22(8):851–860. 10.1038/nm.4123 27376576 10.1038/nm.4123 PMC4935930

[CR41] Joshi VB, Gutierrez Ruiz OL, Razidlo GL (2023) The cell biology of metastatic invasion in pancreatic cancer: updates and mechanistic insights. Cancers 15(7):2169. 10.3390/cancers15072169 37046830 10.3390/cancers15072169 PMC10093482

[CR42] Kai F, Drain AP, Weaver VM (2019) The extracellular matrix modulates the metastatic journey. Dev Cell 49(3):332–346. 10.1016/j.devcel.2019.03.026 31063753 10.1016/j.devcel.2019.03.026 PMC6527347

[CR43] Kalluri R, LeBleu VS (2020) The biology, function, and biomedical applications of exosomes. Science 367(6478):eaau6977. 10.1126/science.aau6977 32029601 10.1126/science.aau6977 PMC7717626

[CR44] Kamphorst JJ, Nofal M, Commisso C, Hackett SR, Lu W, Grabocka E et al (2015) Human pancreatic cancer tumors are nutrient poor and tumor cells actively scavenge extracellular protein. Can Res 75(3):544–553. 10.1158/0008-5472.can-14-2211 10.1158/0008-5472.CAN-14-2211 PMC4316379 25644265

[CR45] Kim PK, Halbrook CJ, Kerk SA, Radyk M, Wisner S, Kremer DM et al (2021) Hyaluronic acid fuels pancreatic cancer cell growth. eLife 10:e62645. 10.7554/eLife.62645 34951587 10.7554/eLife.62645 PMC8730721

[CR46] Kleeff J, Korc M, Apte M, La Vecchia C, Johnson CD, Biankin AV et al (2016) Pancreatic cancer. Nat Rev Dis Primers 2(1):16022. 10.1038/nrdp.2016.22 27158978 10.1038/nrdp.2016.22

[CR47] Kleinman HK, Martin GR (2005) Matrigel: basement membrane matrix with biological activity. Semin Cancer Biol 15(5):378–386. 10.1016/j.semcancer.2005.05.004 15975825 10.1016/j.semcancer.2005.05.004

[CR48] Kojima H, Kushige H, Yagi H, Nishijima T, Moritoki N, Nagoshi N et al (2023) Combinational treatment involving decellularized extracellular matrix hydrogels with mesenchymal stem cells increased the efficacy of cell therapy in pancreatitis. Cell Transplant 32:09636897231170437. 10.1177/09636897231170437 37191199 10.1177/09636897231170437 PMC10192953

[CR49] Kuiper JW, van Horssen R, Oerlemans F, Peters W, van Dommelen MM, te Lindert MM et al (2009) Local ATP generation by brain-type creatine kinase (CK-B) facilitates cell motility. PLoS ONE 4(3):e5030. 10.1371/journal.pone.0005030 19333390 10.1371/journal.pone.0005030 PMC2659440

[CR50] Kumar K, Kanojia D, Bentrem DJ, Hwang RF, Butchar JP, Tridandapani S et al (2023) Targeting BET proteins decreases hyaluronidase-1 in pancreatic cancer. Cells 12(11):1490. 10.3390/cells12111490 37296612 10.3390/cells12111490 PMC10253193

[CR51] Kurmi K, Hitosugi S, Yu J, Boakye-Agyeman F, Wiese EK, Larson TR et al (2018) Tyrosine phosphorylation of mitochondrial creatine kinase 1 enhances a druggable tumor energy shuttle pathway. Cell Metab 28(6):833–847.e8. 10.1016/j.cmet.2018.08.008 30174304 10.1016/j.cmet.2018.08.008 PMC6281770

[CR52] Lachowski D, Cortes E, Pink D, Chronopoulos A, Karim SA, Morton JP et al (2017) Substrate rigidity controls activation and durotaxis in pancreatic stellate cells. Sci Rep 7(1):2506. 10.1038/s41598-017-02689-x 28566691 10.1038/s41598-017-02689-x PMC5451433

[CR53] Laklai H, Miroshnikova YA, Pickup MW, Collisson EA, Kim GE, Barrett AS et al (2016) Genotype tunes pancreatic ductal adenocarcinoma tissue tension to induce matricellular fibrosis and tumor progression. Nat Med 22(5):497–505. 10.1038/nm.4082 27089513 10.1038/nm.4082 PMC4860133

[CR54] Lee KY, Mooney DJ (2001) Hydrogels for tissue engineering. Chem Rev 101(7):1869–1880. 10.1021/cr000108x 11710233 10.1021/cr000108x

[CR55] LeSavage BL, Zhang D, Huerta-López C, Gilchrist AE, Krajina BA, Karlsson K et al (2024) Engineered matrices reveal stiffness-mediated chemoresistance in patient-derived pancreatic cancer organoids. Nat Mater 23(8):1138–1149. 10.1038/s41563-024-01908-x 38965405 10.1038/s41563-024-01908-x PMC13098013

[CR56] Levental KR, Yu H, Kass L, Lakins JN, Egeblad M, Erler JT et al (2009) Matrix crosslinking forces tumor progression by enhancing integrin signaling. Cell 139(5):891–906. 10.1016/j.cell.2009.10.027 19931152 10.1016/j.cell.2009.10.027 PMC2788004

[CR57] Lin FY, Chang CY, Nguyen H, Li H, Fishel ML, Lin CC (2023) Viscoelastic hydrogels for interrogating pancreatic cancer-stromal cell interactions. Mater Today Bio 19:100576. 10.1016/j.mtbio.2023.100576 36816601 10.1016/j.mtbio.2023.100576 PMC9929443

[CR58] Liu H-Y, Korc M, Lin C-C (2018) Biomimetic and enzyme-responsive dynamic hydrogels for studying cell-matrix interactions in pancreatic ductal adenocarcinoma. Biomaterials 160:24–36. 10.1016/j.biomaterials.2018.01.012 29353105 10.1016/j.biomaterials.2018.01.012 PMC5815383

[CR59] Liu C, Zhang Q, Zhu S, Liu H, Chen J (2019) Preparation and applications of peptide-based injectable hydrogels. RSC Adv 9(48):28299–28311. 10.1039/C9RA05934B 35530460 10.1039/c9ra05934b PMC9071167

[CR60] Liu Y, Okesola BO, Osuna de la Peña D, Li W, Lin ML, Trabulo S et al (2024) A self-assembled 3D model demonstrates how stiffness educates tumor cell phenotypes and therapy resistance in pancreatic cancer. Adv Healthc Mater 13(17):e2301941. 10.1002/adhm.202301941 38471128 10.1002/adhm.202301941 PMC11468796

[CR61] Lyssiotis CA, Kimmelman AC (2017) Metabolic interactions in the tumor microenvironment. Trends Cell Biol 27(11):863–875. 10.1016/j.tcb.2017.06.003 28734735 10.1016/j.tcb.2017.06.003 PMC5814137

[CR62] Ma Y, Han T, Yang Q, Wang J, Feng B, Jia Y et al (2021) Viscoelastic cell microenvironment: hydrogel-based strategy for recapitulating dynamic ECM mechanics. Adv Funct Mater 31(24):2100848. 10.1002/adfm.202100848

[CR63] Malinova A, Veghini L, Real FX, Corbo V (2021) Cell lineage infidelity in PDAC progression and therapy resistance. Front Cell Dev Biol 9:795251. 10.3389/fcell.2021.795251 34926472 10.3389/fcell.2021.795251 PMC8675127

[CR64] Marstrand-Daucé L, Lorenzo D, Chassac A, Nicole P, Couvelard A, Haumaitre C (2023) Acinar-to-ductal metaplasia (ADM): on the road to pancreatic intraepithelial neoplasia (PanIN) and pancreatic cancer. Int J Mol Sci 24(12):9946. 10.3390/ijms24129946 37373094 10.3390/ijms24129946 PMC10298625

[CR65] Matsuda Y, Furukawa T, Yachida S, Nishimura M, Seki A, Nonaka K et al (2017) The prevalence and clinicopathological characteristics of high-grade pancreatic intraepithelial neoplasia: autopsy study evaluating the entire pancreatic parenchyma. Pancreas 46(5):658–664. 10.1097/mpa.0000000000000786 28196020 10.1097/MPA.0000000000000786

[CR66] Mattheolabakis G, Milane L, Singh A, Amiji MM (2015) Hyaluronic acid targeting of CD44 for cancer therapy: from receptor biology to nanomedicine. J Drug Target 23(7–8):605–618. 10.3109/1061186x.2015.1052072 26453158 10.3109/1061186X.2015.1052072

[CR67] Mecham RP (2012) Overview of extracellular matrix. Curr Protoc Cell Biol. Chapter 10:10.1.1-.1.6. 10.1002/0471143030.cb1001s00 10.1002/0471143030.cb1001s00 18228295

[CR68] Menezes S, Okail MH, Jalil SMA, Kocher HM, Cameron AJM (2022) Cancer-associated fibroblasts in pancreatic cancer: new subtypes, new markers, new targets. J Pathol 257(4):526–544. 10.1002/path.5926 35533046 10.1002/path.5926 PMC9327514

[CR69] Miao X, Wang Y, Miao Z, Pan H (2022) A comprehensive review of the progress of cell migration inducing hyaluronidase 1. Medicine 101(47):e31610. 10.1097/MD.0000000000031610 36451490 10.1097/MD.0000000000031610 PMC9704909

[CR70] Mierke CT (2024) Extracellular matrix cues regulate mechanosensing and mechanotransduction of cancer cells. Cells 13(1):96. 10.3390/cells13010096 38201302 10.3390/cells13010096 PMC10777970

[CR71] Moridani MY, Bromberg IL (2003) Lipase and pancreatic amylase versus total amylase as biomarkers of pancreatitis: an analytical investigation. Clin Biochem 36(1):31–33. 10.1016/s0009-9120(02)00419-8 12554057 10.1016/s0009-9120(02)00419-8

[CR72] Nakamura S, Sadakari Y, Ohtsuka T, Okayama T, Nakashima Y, Gotoh Y et al (2019) Pancreatic juice exosomal MicroRNAs as biomarkers for detection of pancreatic ductal adenocarcinoma. Ann Surg Oncol 26(7):2104–2111. 10.1245/s10434-019-07269-z 30820789 10.1245/s10434-019-07269-z

[CR73] Nguyen HD, Lin C-C (2024) Viscoelastic stiffening of gelatin hydrogels for dynamic culture of pancreatic cancer spheroids. Acta Biomater 177:203–215. 10.1016/j.actbio.2024.02.010 38354874 10.1016/j.actbio.2024.02.010 PMC10958777

[CR74] Olive KP, Jacobetz MA, Davidson CJ, Gopinathan A, McIntyre D, Honess D et al (2009) Inhibition of Hedgehog signaling enhances delivery of chemotherapy in a mouse model of pancreatic cancer. Science 324(5933):1457–1461. 10.1126/science.1171362 19460966 10.1126/science.1171362 PMC2998180

[CR75] Papadakos SP, Dedes N, Pergaris A, Gazouli M, Theocharis S (2022) Exosomes in the treatment of pancreatic cancer: a moonshot to PDAC treatment? Int J Mol Sci 23(7):3620. 10.3390/ijms23073620 35408980 10.3390/ijms23073620 PMC8998433

[CR76] Papalazarou V, Zhang T, Paul NR, Juin A, Cantini M, Maddocks ODK et al (2020) The creatine–phosphagen system is mechanoresponsive in pancreatic adenocarcinoma and fuels invasion and metastasis. Nat Metab 2(1):62–80. 10.1038/s42255-019-0159-z 32694686 10.1038/s42255-019-0159-z PMC7617069

[CR77] Papalazarou V, Drew J, Juin A, Spence HJ, Whitelaw J, Nixon C, et al (2022) Collagen VI expression is negatively mechanosensitive in pancreatic cancer cells and supports the metastatic niche. J Cell Sci 135(24). 10.1242/jcs.259978 10.1242/jcs.259978 PMC9845737 36546396

[CR78] Paul CD, Hung W-C, Wirtz D, Konstantopoulos K (2016) Engineered models of confined cell migration. Annu Rev Biomed Eng 18:159–180. 10.1146/annurev-bioeng-071114-040654 27420571 10.1146/annurev-bioeng-071114-040654 PMC5369412

[CR79] Petersen GM (2016) Familial pancreatic cancer. Semin Oncol 43(5):548–553. 10.1053/j.seminoncol.2016.09.002 27899186 10.1053/j.seminoncol.2016.09.002 PMC5234085

[CR80] Petrov MS, Yadav D (2019) Global epidemiology and holistic prevention of pancreatitis. Nat Rev Gastroenterol Hepatol 16(3):175–184. 10.1038/s41575-018-0087-5 30482911 10.1038/s41575-018-0087-5 PMC6597260

[CR81] Piccolo S, Panciera T, Contessotto P, Cordenonsi M (2023) YAP/TAZ as master regulators in cancer: modulation, function and therapeutic approaches. Nat Cancer 4(1):9–26. 10.1038/s43018-022-00473-z 36564601 10.1038/s43018-022-00473-z PMC7614914

[CR82] Piffoux M, Eriau E, Cassier PA (2021) Autophagy as a therapeutic target in pancreatic cancer. Br J Cancer 124(2):333–344. 10.1038/s41416-020-01039-5 32929194 10.1038/s41416-020-01039-5 PMC7852577

[CR83] Ping J, Qi L, Wang Q, Liu S, Jiang Y, Yu L et al (2021) An integrated liquid crystal sensing device assisted by the surfactant-embedded smart hydrogel. Biosens Bioelectron 187:113313. 10.1016/j.bios.2021.113313 33989909 10.1016/j.bios.2021.113313

[CR84] Provenzano PP, Cuevas C, Chang AE, Goel VK, Von Hoff DD, Hingorani SR (2012) Enzymatic targeting of the stroma ablates physical barriers to treatment of pancreatic ductal adenocarcinoma. Cancer Cell 21(3):418–429. 10.1016/j.ccr.2012.01.007 22439937 10.1016/j.ccr.2012.01.007 PMC3371414

[CR85] Rahib L, Smith BD, Aizenberg R, Rosenzweig AB, Fleshman JM, Matrisian LM (2014) Projecting cancer incidence and deaths to 2030: the unexpected burden of thyroid, liver, and pancreas cancers in the United States. Cancer Res 74(11):2913–2921. 10.1158/0008-5472.can-14-0155 24840647 10.1158/0008-5472.CAN-14-0155

[CR86] Rahib L, Wehner MR, Matrisian LM, Nead KT (2021) Estimated projection of US Cancer incidence and death to 2040. JAMA Netw Open. 4(4):e214708. 10.1001/jamanetworkopen.2021.4708 33825840 10.1001/jamanetworkopen.2021.4708 PMC8027914

[CR87] Rath N, Olson MF (2012) Rho-associated kinases in tumorigenesis: re-considering ROCK inhibition for cancer therapy. EMBO Rep 13(10):900–908. 10.1038/embor.2012.127 22964758 10.1038/embor.2012.127 PMC3463970

[CR88] Rice AJ, Cortes E, Lachowski D, Cheung BCH, Karim SA, Morton JP et al (2017) Matrix stiffness induces epithelial–mesenchymal transition and promotes chemoresistance in pancreatic cancer cells. Oncogenesis 6(7):e352. 10.1038/oncsis.2017.54 28671675 10.1038/oncsis.2017.54 PMC5541706

[CR89] Richards KE, Zeleniak AE, Fishel ML, Wu J, Littlepage LE, Hill R (2017) Cancer-associated fibroblast exosomes regulate survival and proliferation of pancreatic cancer cells. Oncogene 36(13):1770–1778. 10.1038/onc.2016.353 27669441 10.1038/onc.2016.353 PMC5366272

[CR90] Rubiano A, Delitto D, Han S, Gerber M, Galitz C, Trevino J et al (2018) Viscoelastic properties of human pancreatic tumors and in vitro constructs to mimic mechanical properties. Acta Biomater 67:331–340. 10.1016/j.actbio.2017.11.037 29191507 10.1016/j.actbio.2017.11.037 PMC5797706

[CR91] Sato N, Kohi S, Hirata K, Goggins M (2016) Role of hyaluronan in pancreatic cancer biology and therapy: once again in the spotlight. Cancer Sci 107(5):569–75. 10.1111/cas.12913 26918382 10.1111/cas.12913 PMC4970823

[CR92] Shi J, Deng Q, Wan C, Zheng M, Huang F, Tang B (2017) Fluorometric probing of the lipase level as acute pancreatitis biomarkers based on interfacially controlled aggregation-induced emission (AIE). Chem Sci 8(9):6188–6195. 10.1039/C7SC02189E 28989651 10.1039/c7sc02189e PMC5628346

[CR93] Shichi Y, Gomi F, Sasaki N, Nonaka K, Arai T, Ishiwata T (2022) Epithelial and mesenchymal features of pancreatic ductal adenocarcinoma cell lines in two- and three-dimensional cultures. J Pers Med 12(5):746. 10.3390/jpm12050746 35629168 10.3390/jpm12050746 PMC9146102

[CR94] Steer Michael L, Waxman I, Freedman S (1995) Chronic Pancreatitis. N Engl J Med 332(22):1482–1490. 10.1056/nejm199506013322206 7739686 10.1056/NEJM199506013322206

[CR95] Szatmary P, Grammatikopoulos T, Cai W, Huang W, Mukherjee R, Halloran C et al (2022) Acute pancreatitis: diagnosis and treatment. Drugs 82(12):1251–1276. 10.1007/s40265-022-01766-4 36074322 10.1007/s40265-022-01766-4 PMC9454414

[CR96] Tian C, Clauser KR, Öhlund D, Rickelt S, Huang Y, Gupta M et al (2019) Proteomic analyses of ECM during pancreatic ductal adenocarcinoma progression reveal different contributions by tumor and stromal cells. Proc Natl Acad Sci U S A 116(39):19609–19618. 10.1073/pnas.1908626116 31484774 10.1073/pnas.1908626116 PMC6765243

[CR97] Tse JR, Engler AJ (2010) Preparation of hydrogel substrates with tunable mechanical properties. Curr Protoc Cell Biol 47(1):10–16. 10.1002/0471143030.cb1016s47 10.1002/0471143030.cb1016s47 20521229

[CR98] Walker M, Luo J, Pringle EW, Cantini M (2021) ChondroGELesis: hydrogels to harness the chondrogenic potential of stem cells. Mater Sci Eng, C 121:111822. 10.1016/j.msec.2020.111822 10.1016/j.msec.2020.111822 33579465

[CR99] Walker M, Pringle EW, Ciccone G, Oliver-Cervelló L, Tassieri M, Gourdon D et al (2024) Mind the viscous modulus: the mechanotransductive response to the viscous nature of isoelastic matrices regulates stem cell chondrogenesis. Adv Healthcare Mater 13(9):2302571. 10.1002/adhm.202302571 10.1002/adhm.202302571 PMC11481034 38014647

[CR100] Wang D, Li Y, Ge H, Ghadban T, Reeh M, Güngör C (2022) The extracellular matrix: a key accomplice of cancer stem cell migration, metastasis formation, and drug resistance in PDAC. Cancers (Basel) 14(16):3998. 10.3390/cancers14163998 36010993 10.3390/cancers14163998 PMC9406497

[CR101] Xiao W, Pahlavanneshan M, Eun C-Y, Zhang X, DeKalb C, Mahgoub B et al (2022) Matrix stiffness mediates pancreatic cancer chemoresistance through induction of exosome hypersecretion in a cancer associated fibroblasts-tumor organoid biomimetic model. Matrix Biol Plus 14:100111. 10.1016/j.mbplus.2022.100111 35619988 10.1016/j.mbplus.2022.100111 PMC9126837

[CR102] Yeldag G, Rice A, Del Río Hernández A (2018) Chemoresistance and the self-maintaining tumor microenvironment. Cancers (Basel) 10(12):471. 10.3390/cancers10120471 30487436 10.3390/cancers10120471 PMC6315745

[CR103] Ying H, Kimmelman AC, Lyssiotis CA, Hua S, Chu GC, Fletcher-Sananikone E et al (2012) Oncogenic Kras maintains pancreatic tumors through regulation of anabolic glucose metabolism. Cell 149(3):656–670. 10.1016/j.cell.2012.01.058 22541435 10.1016/j.cell.2012.01.058 PMC3472002

[CR104] Zhao F, Zhang L, Wei M, Duan W, Wu S, Kasim V (2022) Mechanosensitive ion channel PIEZO1 signaling in the hall-marks of cancer: structure and functions. Cancers 14(19):4955. 10.3390/cancers14194955 36230880 10.3390/cancers14194955 PMC9563973

[CR105] Zhou S, Li X-N (2015) The progress of research about pancreatic lipase. Sheng Li Ke Xue Jin Zhan 46(1):6–10. https://europepmc.org/article/med/26103719 26103719

[CR106] Zhou Y, Pan M, Lin R, Huang H (2024) Advances in hydrogel materials applied to pancreatic-related diseases. J Pancreatol 7(3):222–232. 10.1097/JP9.0000000000000158

[CR107] Zhu J (2010) Bioactive modification of poly(ethylene glycol) hydrogels for tissue engineering. Biomaterials 31(17):4639–4656. 10.1016/j.biomaterials.2010.02.044 20303169 10.1016/j.biomaterials.2010.02.044 PMC2907908

